# A Spatial Analysis of Rift Valley Fever Virus Seropositivity in Domestic Ruminants in Tanzania

**DOI:** 10.1371/journal.pone.0131873

**Published:** 2015-07-10

**Authors:** Calvin Sindato, Dirk U. Pfeiffer, Esron D. Karimuribo, Leonard E. G. Mboera, Mark M. Rweyemamu, Janusz T. Paweska

**Affiliations:** 1 National Institute for Medical Research, Tabora, Tanzania; 2 Department of Veterinary Medicine and Public Health, Sokoine University of Agriculture, Morogoro, Tanzania; 3 Southern African Centre for Infectious Disease Surveillance, Morogoro, Tanzania; 4 Veterinary Epidemiology, Economics & Public Health Group, Royal Veterinary College, London, United Kingdom; 5 National Institute for Medical Research, Dar es Salaam, Tanzania; 6 Center for Emerging and Zoonotic Diseases, National Institute for Communicable Diseases, National Health Laboratory Service, Sandringham, South Africa; 7 School of Pathology, Faculty of Health Sciences, University of the Witwatersrand, Johannesburg, South Africa; The University of Texas Medical Branch, UNITED STATES

## Abstract

Rift Valley fever (RVF) is an acute arthropod-borne viral zoonotic disease primarily occurring in Africa. Since RVF-like disease was reported in Tanzania in 1930, outbreaks of the disease have been reported mainly from the eastern ecosystem of the Great Rift Valley. This cross-sectional study was carried out to describe the variation in RVF virus (RVFV) seropositivity in domestic ruminants between selected villages in the eastern and western Rift Valley ecosystems in Tanzania, and identify potential risk factors. Three study villages were purposively selected from each of the two Rift Valley ecosystems. Serum samples from randomly selected domestic ruminants (n = 1,435) were tested for the presence of specific immunoglobulin G (IgG) and M (IgM), using RVF enzyme-linked immunosorbent assay methods. Mixed effects logistic regression modelling was used to investigate the association between potential risk factors and RVFV seropositivity. The overall RVFV seroprevalence (n = 1,435) in domestic ruminants was 25.8% and speciesspecific seroprevalence was 29.7%, 27.7% and 22.0% in sheep (n = 148), cattle (n = 756) and goats (n = 531), respectively. The odds of seropositivity were significantly higher in animals sampled from the villages in the eastern than those in the western Rift Valley ecosystem (OR = 1.88, CI: 1.41, 2.51; p<0.001), in animals sampled from villages with soils of good than those with soils of poor water holding capacity (OR = 1.97; 95% CI: 1.58, 3.02; p< 0.001), and in animals which had been introduced than in animals born within the herd (OR = 5.08, CI: 2.74, 9.44; p< 0.001). Compared with animals aged 1–2 years, those aged 3 and 4–5 years had 3.40 (CI: 2.49, 4.64; p< 0.001) and 3.31 (CI: 2.27, 4.82, p< 0.001) times the odds of seropositivity. The findings confirm exposure to RVFV in all the study villages, but with a higher prevalence in the study villages from the eastern Rift Valley ecosystem.

## Introduction

**Fig 1:**
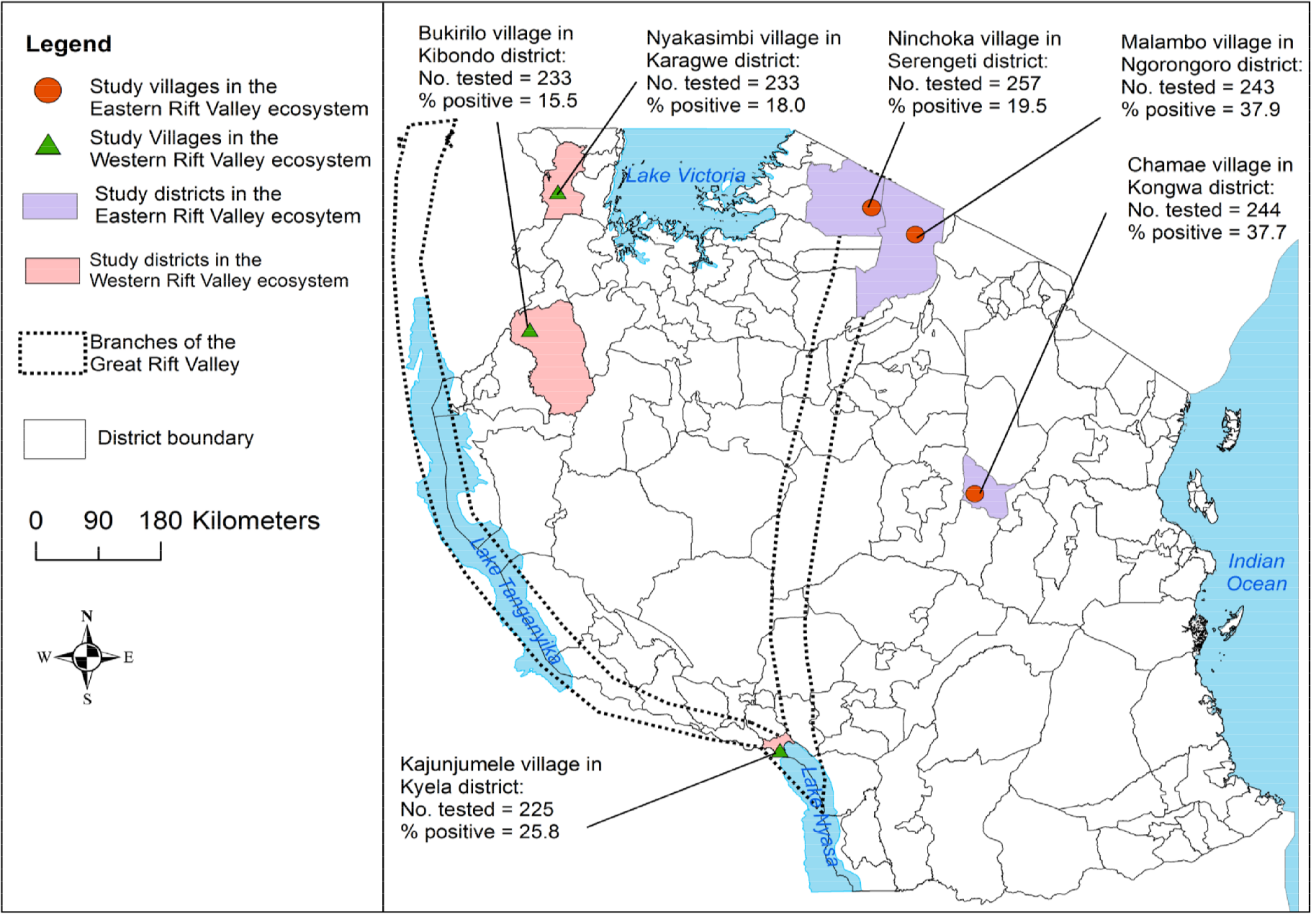
Map of Tanzania showing locations of study villages and districts. Overall, higher RVFV seropositivity was recorded in domestic ruminants from the villages located in the eastern than those in the western Rift Valley ecosystem.

Rift Valley fever (RVF) is an acute vector borne zoonotic viral disease that is caused by RVF virus (RVFV) belonging to the genus *Phlebovirus* of family *Bunyaviridae* [*1, 2*]. It presents a potential threat to the human and animal population mainly in Africa [*1–4*]. The disease is named after the Great Rift Valley system of East Africa, where RVFV was first isolated in 1931 during an outbreak of abortions and deaths in exotic wool sheep in Kenya after heavy rainfall [*5*]. The Great Rift Valley system is a long depression in the earth that runs down the eastern side of Africa. It extends from Syria in the Middle East, right down to Mozambique in south-eastern Africa [*6*]. Tanzania has two branches of this system, forming the eastern and western ecosystems (Fig 1).

RVF affects sheep, goats and cattle causing abortion in females and a high mortality rate in newborn animals leading to considerable economic losses [*7, 8*]. Exposure of humans occurs through direct contact with blood or aborted materials from infected animals, or through mosquito bites [*9*]. Clinical disease in humans presents as mild to moderate severe influenza-like illness that may be complicated by ocular lesions, encephalitis or a fatal haemorrhagic state [*9*]. Although the mechanisms of virus maintenance during inter-epidemic periods (IEP) are poorly understood, it is believed that RVFV is maintained during this time in aedine mosquito eggs, and outbreaks occur following abnormally heavy rains that cause flooding, especially in swampy areas. This results in an increase in the mosquito population that acts as primary and secondary transmitter of the disease [*10*].

Eastern and southern African countries have reported several RVF outbreaks that resulted in substantial losses amongst animals and humans [*11–13*]. During the last large outbreak in 2006/07 in Tanzania, Kenya and Somalia, more than 1,000 cases and 323 deaths were reported in humans [*11*]. In 2011, South Africa was severely affected by the disease [*14*]. RVF outbreaks have also been reported in Zambia in 1985 [*15*], Senegal in 1987 [*16*], Egypt in 2003 [*17*], Madagascar in 2008 [*18*], Mauritania in 2010 [*19*], Sudan in 2010 [*20, 21*], and Namibia, 2010 [*22*]. In 2001–2002 RVF outbreaks were reported beyond Africa in Saudi Arabia and Yemen [*23–26*]. In 2007, the disease was also reported in Mayotte [*27*]. The potential for further geographical spread to areas that have never reported outbreaks of the disease has been suggested [*28–30*].

In Tanzania, RVF has been a notifiable disease under both the country Animal Disease Act since 1980 [*31*] and the Integrated Disease Surveillance and Response (IDSR) Guidelines of the Ministry of Health and Social Welfare since 2011 [*32*]. Since RVF-like disease was first reported in domestic ruminants in Tanzania in 1930, the majority (71.43%, n = 14) of the regions in the eastern ecosystem of the Great Rift Valley had reported RVF outbreaks in the past compared with a smaller proportion (27.27%, n = 11) of regions in the western ecosystem [*33–35*]. Thus far, epidemiological RVF research in Tanzania has been limited to outbreak investigations, and limited data exist about RVFV activity during the inter-epidemic period (IEP) in locations with and without history of outbreaks [*36–38*]. Because of the absence of effective surveillance systems and the fact that RVF outbreaks typically occur in remote locations, it is believed that the disease is underreported in Tanzania. In addition, during the IEP, RVF tends to express itself through low level clinical and/or subclinical incidence, which is unlikely to be detected or reported by farmers and/or livestock officers or it may be misdiagnosed as other diseases with similar clinical manifestations. The low incidence of the dramatic clinical manifestations of RVFV infections has made it difficult to rely on reports based on the clinical case definition for estimating the full extent of RVFV infection during the IEP.

Knowledge about the exposure status to RVFV, the levels of herd immunity and the factors influencing local maintenance of infection and its spatial spread is important for developing strategic interventions such as animal vaccination and public health education programmes. Serological surveillance has been found to be an effective active monitoring tool for determining infection levels and the extent of herd immunity. Evidence of prior exposure to RVFV can be obtained by enzyme linked immunosorbent assay (ELISA) for detecting anti-RVFV antibodies [*39–45*]. Highly sensitive and specific capture and inhibition ELISAs have been developed and validated for the detection of specific immunoglobulin M (IgM) and G (IgG), against RVFV in cattle, sheep, goats and humans [*43*, *44*].

It is unclear why RVF outbreaks have been reported mainly in the eastern rather than the western Rift Valley ecosystem in Tanzania. Probably past disease surveillance activities focussed more on the eastern rather than the western Rift Valley ecosystem. It is likely that there is no variation in the levels of RVFV seropositivity in domestic ruminants during the IEP between the two Rift Valley ecosystems in the country. In this study, we examine the plausibility of this hypothesis by describing the spatial variability in RVFV seropositivity based on the prevalence of RVFV-specific IgG and IgM antibodies in domestic ruminants sampled during the IEP amongst a small sample of villages, in the eastern and western ecosystems of the Great Rift Valley in Tanzania. We also analyzed the potential risk factors associated with RVFV seropositivity outcomes in the two Rift Valley ecosystems. This is an essential step towards improving the country disease risk maps for strategic cost-effective usage of limited disease control resources.

## Materials and Methods

### Ethics Statement

This study received ethical approval from the Medical Research Coordinating Committee of the National Institute for Medical Research in Tanzania (ethics certificate number NIMR/HQ/R.8a/Vol.IX/1296). At the time of study implementation within Tanzania there was no dedicated Institutional Animal Care and Use Committee (IACUC) or animal ethics committee to evaluate and approve the protocol specifically related to involvement of livestock in this research. The study protocol was also reviewed by the National Veterinary Epidemiologist at the Ministry of Livestock and Fisheries Development in Tanzania. The present study did not involve endangered or protected species, and the animals used in this study were not sacrificed for research purposes.

Prior to data collection, district veterinary officers and local community leaders in the study areas were informed about the study and they granted permission to conduct this study in their respective areas. The permission to carry out livestock sampling was obtained directly from the owners. The purpose of this study was explained to livestock owners and they were made aware that participation was voluntary and their identity would be kept confidential. They had an opportunity to ask questions, and before participating, participants had provided written informed consent for blood sampling of their livestock and related survey questions. For participants who were unable to read, the text was read and explained to them, and a fingerprint was obtained in front of a literate witness.

Blood collection from livestock was conducted solely for the purposes of this study and it was performed aseptically by a veterinarian who was one of the investigators. Animals were humanely treated during sample collection and clinical examination was conducted in accordance with the World Organization for Animal Health (OIE) guidelines for use of animals in research and education [*46*]. The study was restricted to blood sampling procedures and measurements that did not cause stress, suffering or any harm in animals. To minimize stress and sufferings, animals were physically restrained to prevent any movement that would result in lacerating the blood vessel or any other harm. A single jugular vein puncture was performed to collect 3ml of blood from each study animal. After blood sample collection, digital pressure was applied to the puncture site using sterile gauze until complete haemostasis had been achieved. A completed ARRIVE guidelines checklist is included in S1 ARRIVE Checklist.

### Study Design

A cross-sectional study design was used based on random selection of animals from six villages which were purposively identified representing the spectrum of occurrence risk amongst areas in Tanzania suitable for RVF. Villages had to be considered to be at risk of RVF based on epidemiological knowledge, but did not have to have reported RVF occurrence in the past.

#### Selection of districts and villages

The sampling process involved a two-stage purposive selection of districts and villages in the eastern and western Rift Valley ecosystems based on the model-predicted habitat suitability for RVF occurrence, reporting status of RVF outbreaks and local knowledge about RVF risk in Tanzania. Within each selected village a two stage random sampling process was used to select the herds and animals. The number of villages was not based on statistical considerations, but on logistic and resource factors.

Based on model predictions of habitat suitability for RVFV activity and findings of the past studies [*33–35*], stratification was performed first, for the regions in the country at low, medium and high risk of disease occurrence and second, for the regions with and with no history of RVF outbreaks. In this study, high risk referred to areas with a probability of RVF occurrence that ranged from 0.49–0.87 representing locations in the north-eastern, central and Lake Victoria zones of the country. Medium risk referred to areas with a probability of RVF occurrence that ranged from 0.30–0.48 representing locations in the north-western and southern-highland zones of the country. Low risk referred to areas with a probability of RVF occurrence that ranged from 0.07–0.29 representing locations in the western and southern zones of the country. Three regions were then purposively selected from each of the Rift Valley ecosystem with various levels of risk of disease occurrence. The regions that were selected in the eastern Rift Valley ecosystem were Arusha (high risk), Dodoma (high risk) and Mara (medium risk). In the western Rift Valley ecosystem Mbeya (medium risk), Kigoma (low risk) and Kagera (low risk) were selected.

Within each of these six regions, all district veterinary officers were consulted in order to identify a district within each region considered to be at highest risk of RVF occurrence. Criteria used included presence of shallow depressions/locations that are subject to regular flooding, ecological features suitable for mosquito breeding and survival/experience of mosquito swarms during the rainy season, relatively high concentration of domestic ruminants, proximity to forest, rivers, lakes, wildlife and presence of areas with history of RVF occurrence. The district within the region that was identified to have most of these epidemiological characteristics was selected for the study, even if they had never reported RVF outbreaks. Three of the selected districts are in the eastern Rift Valley ecosystem: Ngorongoro (Arusha region), Kongwa (Dodoma region) and Serengeti (Mara region). The other three selected districts are in the western Rift Valley ecosystem: Kibondo (Kigoma region), Karagwe (Kagera region) and Kyela (Mbeya region) (Fig 1). Within the selected districts, all villages keeping domestic ruminants and not having a history of vaccination against RVF were identified using local official veterinary records. Based on the above criteria for identifying the six study districts, additional discussions were then held with local veterinary/agricultural officers, community leaders and livestock keepers to identify one village within each district that was considered to be at highest risk for RVFV activity. The selected villages were Chamae in Kongwa, Malambo in Ngorongoro, Ninchoka in Serengeti, Bukirilo in Kibondo, Nyakasimbi in Karagwe and Kajunjumele in Kyela (Fig 1).

#### Description of the study districts

This cross-sectional study was conducted in six villages from six districts in Tanzania (Fig 1) between April and August 2013, approximately six years after the last RVF outbreak in the country in 2006–2007. Weather data, elevation, livestock density and history of Rift Valley fever outbreaks in the study districts are presented in Table 1. Kongwa district is at the centre of Tanzania’s network of major roads. Serengeti and Ngorongoro districts are part of the national network of major roads connecting Arusha and Mara regions through the Ngorongoro Conservation Area (NCA) and Serengeti National Park (SENAPA) and then proceeding towards the north to the Kenyan border. Kibondo, Karagwe and Kyela districts are at the periphery of the national network of major roads.

**Table 1:** Soil types, elevation (metres above sea level), weather parameters, livestock density (heads per square kilometre) and history of Rift Valley fever outbreaks (1930 to 2007) in the study areas.

District	Village	Latitude (S)	Longitude (E)	Predominant soil type	Average elevation	Rainfall pattern	Average total annual rainfall mm)	Average monthly rainfall (mm)	Mean minimum temperature (°C)	Mean maximum temperature (°C)	Cattle density	Goats density	Sheep density	No. RVF outbreaks
Ngorongoro	Malambo	-2.45692°	35.16470°	**Phaeozems	1213	Bimodal	688	47	10	32	27	26	14	10
Serengeti	Ninchoka	-1.92961°	34. 54275°	**Alisols	1453	Bimodal	888	73	15	25	30	14	6	2
Kongwa	Chamae	-5.98833°	36.42812°	**Luvisols	1126	Unimodal	712	36	14	30	32	25	8	1
Karagwe	Nyakasimbi	-1.93781°	31.08345°	*Ferralsols	1564	Bimodal	966	74	20	30	19	21	1	0
Kibondo	Bukirilo	-3.26392°	30.72514°	*Ferralsols	1330	Unimodal	1232	61	15	30	2	8	1	0
Kyela	Kajunjumele	-9.62272°	33.91139°	**Fluvisols	486	Unimodal	1270	106	19	31	21	4	2	0

*soils characterized by poor water holding capacity properties

** soils characterized by good water holding capacity properties

#### Sources of data for weather, soil types and history of RVF outbreaks in the study districts

Weather data (average total annual rainfall, average monthly rainfall, and rainfall pattern, mean minimum and maximum temperatures) for the years 1977 to 2012 were obtained from the Tanzania Meteorological Agency (TMA). Data for soil types were obtained from the Mlingano Agricultural Research Institute in Tanga, Tanzania. Data for livestock density was obtained from the Ministry of Livestock and Fisheries Development in Tanzania based on the national sample census of agriculture conducted in 2007/2008. The history of RVF outbreaks in the study areas for the years 1930 to 2007 was obtained from a previous study in Tanzania [*35*].

### Sampling of Animals and Blood Collection

The sampling frame included a list of all herds from the list of livestock keepers in selected villages keeping at least one of the three domestic ruminant species (cattle, sheep and goats). For logistic and resource factors, a target was set to randomly select 20 herds in each village. During discussions with district veterinary officers, it was estimated that majority of herds would be comprised of 5 to 20 domestic ruminants. Therefore, in order to avoid oversampling in larger herds a maximum of 20 ruminant animals were bled (i.e. 10 cattle, 5 goats and 5 sheep depending on the herd size and species composition within the herd at the time of sampling). Blood samples were collected aseptically by jugular vein puncture into labelled vacutainer tubes with clot activator. They were kept in a cool box with ice packs before separating the serum from coagulated whole blood into labelled 1.8 ml Cryovial tubes. They were then stored in -196°C liquid nitrogen gas in the field before being transferred to the laboratory, where they were kept in a freezer at -20°C until laboratory analysis.

In addition to the serum samples, epidemiological data were obtained from the household head or the herder and through clinical examination using a semi-structured questionnaire (S1 Text). The epidemiological data collected included the herd species composition, animal species, breed, sex and age, history of abortions in the past 12 months, animal feeding practices/options, whether an animal was born within the herd or introduced (moved) into the herd from another district and whether an animal was sampled from a district with a history of RVF outbreaks. A clinical examination for signs and symptoms suggestive of RVF was conducted for each sampled animal on the day of the herd visit.

#### Age determination

Animals in the herd were not ear-tagged, but the herdsmen/owners/family members were able to identify each animal by name and number of parities. Records on the age of the animals were rarely available in the surveyed herds. In all herds without proper records, individual animal age was estimated based on the history obtained from the herdsmen/owners/family members in relation to the season and year of birth and number of births for female animals. Other methods which were used to estimate the age of the animal were the dentition [47] and horn-ring techniques (http://en.wikipedia.org/wiki/cattle_age_determination). Furthermore, information was gathered as to whether the animal had been present in the herd during the last RVF outbreak that had occurred following the El Niño–related flooding event in the country in 2006–2007. In addition, information was gathered on the year the animal was introduced (for animals not born within the herd) into the herd from sources outside the sampled district and estimated age/physiological status of sexual maturity and number of parities (for females) during the period of introduction.

#### Laboratory examination of serum samples for IgG and IgM specific to RVFV

The serum samples were tested for the presence of anti-RVF antibodies using two commercial enzyme-linked immunosorbent assay (ELISA) kits according to the manufacturer’s (Biological Diagnostic Supplies Limited, UK) instructions. These included IgM-capture ELISA [43] and inhibition ELISA [44]. Initially, all samples were tested using the RVF inhibition ELISA. Serum samples that were positive on the inhibition ELISA were subjected to IgM-capture ELISA. When using inhibition ELISA, the specific activity of each serum (net optical density {OD}) was calculated by subtracting the non-specific background OD in the wells with mock antigen from the OD in wells with virus antigen. The net OD readings were converted to a percentage inhibition (PI) value using the equation: [100 − (mean net OD of test sample/mean net OD of negative control) ×100]. Internal quality control was performed in accordance with the diagnostic test kit manufacturer’s recommendations. The results were interpreted using the cut-off threshold specified by the manufacturer of the test kit. Serum samples with PI equal to or greater than 41.9, 41.4 and 38.4 were considered seropositive for RVF inhibition in cattle, goats and sheep, respectively. The sensitivity of this ELISA method in cattle, goats and sheep is reported to be 100%, 99.6% and 100%, respectively, and its specificity in cattle, goats and sheep is 99.5%, 99.7% and 99.3%, respectively [44]. When using IgM ELISA, the specific activity of each serum (net optical density {OD}) was calculated by subtracting the non-specific background OD in the wells with mock antigen from the OD in wells with virus antigen. Conversion of net OD readings into percentage positivity (PP) was carried out using the equation: (mean net OD of test sample/mean net OD of high-positive control) × 100. Internal quality control was performed according to the kit manufacturer’s recommendations. Sheep, goat, and bovine sera producing PP values ≥7.9, ≥ 9.5 and ≥ 14.3, respectively, were considered to be positive. Due to the transitory presence of IgM in the sera of RVF infected animals, the sensitivity of this ELISA method is influenced by the time post infection at which specimens are collected for testing. Its sensitivity is reported to be 100% in experimental sheep tested 5–42 days post infection. The specificity of this method in sheep, goats and cattle is 98.7%, 99.7% and 100%, respectively [43].

### Data Analysis

The epidemiological and laboratory results were entered into a Microsoft Excel spreadsheet and then imported into STATA version 12 (Statacorp, College Station, TX, USA) for coding, cleaning and statistical analysis. All electronic data related to this study were stored on a password protected computer with access limited only to authorized personnel. The unit of analysis was individual animal. The analysis focused on domestic ruminants ranging from 1 to 5 years of age (born after the last RVF outbreak in 2006–2007) and having been present in the herd for at least one year. A descriptive analysis was carried out. This was followed by univariable analysis to assess initial association between potential risk factors and the outcome variable defined by RVFV seropositivity. Chi-squared test was used to compare the seroprevalence between the villages in the eastern and western Rift Valley ecosystems or the three regional disease risk groups. Furthermore, chi-squared test was used to assess the association between the seroprevalence and the potential risk factor variables: animal species, sex, age, source, breed, history of abortion, feeding practices, animal has diarrhoea/nasal discharge, average total annual rainfall (mm), average monthly rainfall (mm), rainfall pattern and water holding capacity of soils in the study areas, and whether animal were sampled from district with history of RVF outbreaks.

Mixed effects logistic regression modelling was used to investigate the association between various potential risk factors and the outcome variable defined by RVFV seropositivity. The models included herd as a random effect variable to account for dependence of data from the same herd. The village was used as a stratification variable and therefore forced into the model as a fixed effect variable. To take account of possible nonlinear effects of continuous-scale risk factors on the logit form of the outcome variable, these variables were categorised into three contiguous groups, each representing a third of the observations. The analysis was conducted in two steps. First, all potential risk factors were screened for statistical significance at a p-value of ≤ 0.20 in a mixed effects univariable logistic regression analysis. In the second step, the statistically significant variables were included in a mixed effects multivariable logistic regression analysis based on a forward variable selection approach, utilising the likelihood ratio statistic and a p-value ≤ 0.05. Variables not statistically significant in the univariable analysis, but with a known association with RVFV activity or suspected to be a potential confounder were also included in the multivariable analysis. A factor was considered to have a potential confounding effect if its inclusion in the model resulted in a change of ≥25% in the coefficient estimates of other risk factors included in the model compared to its absence. The variables included in the modelling process were limited to those that did not show significant collinearity using a diagnostic cut-off value for tolerance > 0.1 and variance inflation factor < 10 [*48*]. Interaction terms were then introduced into the model to examine the potential presence of effect modification. The discriminatory ability of the final model was assessed using receiver operating characteristic curves (ROC), and quantified by calculating the area under the curve (AUC) [*49*].

## Results

### Characteristics of the Study Population

A total of 1,435 domestic ruminants from 121 herds in six villages in Tanzania were tested for antibodies against RVFV. About an equal proportion of tested serum samples was collected in livestock from the villages in the eastern (51.9%) and western (48.2%) ecosystems of the Rift Valley, and from locations considered to be in a low risk (32.5%), medium risk (33.6%) and high risk (33.9%) regions. The number of tested serum samples ranged from 225 in Kajunjumele village in Kyela district to 257 in Chamae village in Kongwa district (Table 2). The grazing only option was practiced in five villages while stall-feeding combined with grazing was only reported in Kajunjumele village in Kyela district. Livestock kept in the sampled herds were of indigenous breed and crossbreed (Table 2). The indigenous breeds of cattle were as follows (name of district in parentheses): mainly Ankole in Bukirilo (Kibondo) and Nyakasimbi (Karagwe) villages; Tanzanian short horn zebu, borani and Mpwapwa breeds in Chamae village (Kongwa); Tanzanian short horn zebu in Ninchoka (Serengeti), Malambo (Ngorongoro) and Kajunjumele (Kyela) villages. The indigenous breeds of goats and sheep kept in the sampled villages were mainly the Small East African goat and a mix of Red Maasai and Black head Persian (BHP) sheep.

**Table 2:** Number of animals sampled in the study villages and demographic characteristics of the study population.

Village	District	No. animals sampled (%)	Species			Breed		Sex		Median age (years)	Age categories (years)			Animal source	
			Goat (%)	Sheep (%)	Cattle (%)	Indigenous (%)	Cross- breed (%)	Male (%)	Female (%)		1–2 (%)	3 (%)	4–5 (%)	Born within herd (%)	Introduced to the herd (%)
Malambo	Ngorongoro	243 (16.9)	90 (37.0)	68 (28.0)	85 (35)	215 (88.5)	28 (11.5)	59 (24.3)	184 (75.7)	3	77 (31.7)	94 (38.7)	72 (29.6)	210 (86.4)	33 (13.6)
Ninchoka	Serengeti	257 (17.9)	105 (40.8)	32 (12.5)	120 (46.7)	256 (99.6)	1 (0.4)	65 (25.3)	192 (74.7)	2	132 (51.4)	89 (34.6)	36 (14.0)	255 (99.2)	2 (0.8)
Chamae	Kongwa	244 (17.0)	91 (37.3)	29 (11.9)	124 (50.8)	220 (90.2)	24 (9.8)	56 (23)	188 (77)	3	98 (40.2)	126 (51.6)	20 (8.2)	242 (99.2)	2 (0.8)
Nyakasimbi	Karagwe	233 (16.3)	94 (40.3)	8 (3.5)	131 (56.2)	229 (98.3)	4 (1.7)	34 (14.6)	199 (85.4)	3	88 (37.8)	117 (50.2)	28 (12.0)	226 (97.0)	7 (3.0)
Bukirilo	Kibondo	233 (16.3)	137 (58.8)	11 (4.7)	85 (36.5)	229 (98.3)	4 (1.7)	35 (15.0)	198 (85.0)	2	151 (64.8)	41 (17.6)	41 (17.6)	225 (96.6)	8 (3.4)
Kajunjumele	Kyela	225 (15.7)	14 (6.2)	0 (0)	211 (93.8)	225 (100)	0 (0)	50 (22.2)	175 (77.8)	3	53 (23.6)	111 (49.3)	61 (27.1)	225 (100)	0 (0)
**Total**		**1435 (100)**	**531 (37.0)**	**148 (10.3)**	**756 (52.7)**	**1374 (95.7)**	**61 (4.3)**	**299 (20.8)**	**1136 (79.2)**		**599 (41.7)**	**578 (40.3)**	**258 (18.0)**	**1383 (96.4)**	**52 (3.6)**

The majority (95.7%) of tested serum samples were from animals of indigenous breeds (Table 2). The majority of crossbreed animals were sampled in Malambo (Ngorongoro district) and Chamae (Kongwa district) villages in the eastern Rift Valley ecosystem (Table 2). The majority (96.4%) of tested serum samples were from animals born within the sampled herds. More than half of crossbreed animals sampled (52.5%, n = 61) had been introduced into the herd compared with only 1.5% (n = 1374) of the indigenous breeds that had been introduced into the herd (p = 0.001).

The largest number of the introduced animals was sampled from Malambo village in Ngorongoro district (Table 2). All animals sampled in Kajunjumele village in Kyela district were of indigenous breeds (Table 2) and all were born within herds sampled. Amongst the introduced animals, cattle and goats represented 36.5% each and sheep were 27%. The number of specific domestic animals by ruminant species introduced to Malambo village in Ngorongoro district was: sheep (14), goats (11) and cattle (8); in Bukirilo village in Kibondo district was: goats (7) and cattle (1), in Nyakasimbi village in Karagwe district was: cattle (6) and goats (1), in Chamae village in Kongwa district and in Ninchoka village in Serengeti district was: cattle (2 each). The sources for introduced animals were livestock auction markets (70.7%), bride dowry (25.3%) and purchase from a neighbouring country (4.0%). The majority (82.0%, n = 1435) of the sampled animals were aged 1–3 years old and few were aged 4–5 years old (18.0%). At the time of the study, a larger proportion of sampled animals born within herd (42.5%, n = 1383) was 1–2 years old, and 39.5% and 18.8% of them were 3 and 4–5 years old, respectively. Conversely, a larger proportion of introduced animals sampled (61.5%, n = 52) was 3 years old, and 21.2% and 17.3% of them were 1–2 and 4–5 years old, respectively. Overall, the median age of the sampled animals was 3 years of age (range = 1–5 years) and it varied significantly between the study villages (p = 0.0001). The median age of the sampled animals in Bukirilo (Kibondo district) and Ninchoka (Serengeti district) villages was 2 (range = 1–5) while it was 3 (range = 1–5) in the other villages. Cattle accounted for 76.0%, 62.1% and 33.4% of 258 animals aged 4–5 years, 578 animals aged 3 years and 599 animals aged 1–2 years, respectively (p = 0.001). However, there were no reliable records to estimate the age of animals at the time of introduction into the herd.

Of the tested serum samples, 1,136 (79.2%) were from females (Table 2), of which 1.1% had a history of abortion in the past 12 months. No livestock owners and/or herders could recall having ever seen in the herds in the past 12 months signs suggestive of RVF such as massive death of new born/neonatal animals. At the time of the study, all sampled animals had no signs and/or symptoms suggestive of RVF. However, a small proportion of animals presented with diarrhoea (4.4%), nasal discharge (1.2%) and contemporaneous diarrhoea and nasal discharge (0.4%). About half (52.7%) of 1435 serum samples tested were collected from cattle (Table 2). IgG specific to RVFV were detected in all species sampled. IgM specific to RVFV was not detected in any sample. The analyses in this study were therefore based on the IgG sero-status outcomes.

The overall seroprevalence of IgG specific to RVFV was 25.8% (n = 1435) and the specific species seroprevalence was 29.7% (n = 148) in sheep, 27.8% (n = 756) in cattle and 21.9% (n = 531) in goats (p = 0.029) (the range of Inhibition and IgM detection ELISA values for domestic ruminant serum samples tested for antibodies specific to RVFV is presented in S1 Table). A significantly higher proportion of IgG seropositivity was recorded in animals sampled from the three villages in the eastern (31.5%, n = 744) than the three villages in the western (19.7%, n = 691) ecosystem of the Rift Valley (p<0.001).

## Risk Factor Analysis

### Univariable Mixed Effects Logistic Regression Analysis

The results of univariable mixed effects logistic regression analysis are presented in Table 3. RVFV seropositivity varied significantly between the study villages from the two Rift Valley ecosystems. Animals sampled from the three villages in the eastern Rift Valley ecosystem had relatively higher odds of seropositivity compared with their three counterparts in the western Rift Valley ecosystem (OR = 1.88, CI: 1.41, 2.51 p<0.001). The univariable mixed effects data analysis showed that the crossbreed animals had significantly higher odds of IgG seropositivity than indigenous animals. The animals aged 4–5 years had higher odds of RVFV seropositivity than animals aged 1–3 years. Animals that had history of abortion had four times the odds of seropositivity as the animals which had no reports of abortion. This analysis suggested further the animals that had been introduced had five times the odds of seropositivity as the animals born within herd. Animals sampled from villages within the districts where RVF outbreak had been reported had higher odds of seropositivity compared with animals sampled from villages in districts with no reports of RVF outbreaks. Animals from the villages in districts which received total annual rainfall ≤ 712 mm had lower odds for seropositivity, than animals from villages in districts which received more rainfall. Animals sampled from the villages in districts with soil types of good water holding capacity had higher odds of seropositivity than their counterpart from villages within the districts with soils of poor water holding capacity. With respect to livestock density, cattle sampled from villages within the districts with >30 heads of cattle per square kilometre had higher odds of seropositivity than cattle sampled from areas with ≤30 heads of cattle per square kilometre; goats sampled from the villages within the districts with >25 heads of goats per square kilometre had higher odds of seropositivity than goats sampled from areas with ≤ 25 heads of goats per square kilometre and sheep sampled from villages within the districts with >8 heads of goats per square kilometre had higher odds of seropositivity than sheep sampled from areas with ≤ 8 heads of goats per square kilometre.

**Table 3:** Univariable mixed effects logistic regression analysis of risk factors associated with Rift Valley fever seropositivity in domestic ruminants (herd was included as random effect variable).

Variable	No. tested	No. seropositive (%)	OR	95% CI	z value	p value	* Pearson Chi square	*DF	*p value
**Rift Valley ecosystem**									
Study villages in the western Rift Valley ecosystem	691	136	1.0	Ref.	Ref.	Ref.	Ref.		
Study villages in the eastern Rift Valley ecosystem	744	234	1.88	1.41, 2.51	4.29	< 0.001	18.42	1	<0.0001
**Village**									
Bukirilo	233	36 (15.5)	1.00	Ref.	Ref.	Ref.			
Chamae	244	92 (37.7)	3.32	2.13, 5.17	5.29	< 0.001			
Malambo	243	92 (37.9)	3.33	2.15, 5.19	5.31	< 0.001			
Kajunjumele	225	58 (25.8)	1.9	1.19, 3.04	2.69	0.007			
Ninchoka	257	50 (19.5)	1.32	0.83, 2.13	1.15	0.249			
Nyakasimbi	233	42 (18.0)	1.2	0.74, 1.96	0.73	0.463	58.83	5	< 0.001
**Rainfall pattern in the study areas**									
Unimodal	702	186 (26.5)	1.00	Ref.	Ref.	Ref.			
Bimodal	733	184 (25.1)	0.91	0.67–1.24	-0.60	0.551	0.36	1	0.551
**Average total annual rainfall (mm) in the study areas**									
≤712	487	184 (37.7)	1.00	Ref.	Ref.	Ref.			
>712–966	490	92 (18.8)	0.38	0.28, 0.51	-6.26	<0.001			
>966	458	94 (20.5)	0.43	0.31, 0.58	-5.53	<0.001	49.97	2	<0.001
**Average monthly rainfall (mm) in the study areas**									
≤47	487	184 (37.8)	1.00	Ref.	Ref.	Ref.			
>47–74	490	87 (17.6)	0.35	0.26, 0.47	-6.76	< 0.001			
>74	458	100 (21.8)	0.46	0.34, 0.62	-5.15	< 0.001	52.87	2	< 0.001
**Water holding capacity of soils in the study areas**									
Poor	466	78 (16.7)	1.00	Ref.	Ref.	Ref.			
Good	969	292 (30.1)	2.18	1.58, 3.02	4.73	< 0.001	22.36	1	< 0.001
**Species**									
Caprine	531	116 (21.9)	1.00	Ref.	Ref.	Ref.			
Ovine	148	44 (29.7)	1.51	0.96, 2.38	1.78	0.075			
Bovine	756	210 (27.8)	1.39	1.01, 1.91	2.02	0.044	5.32	2	0.071
**Sex**									
Male	299	64 (21.4)	1.00	Ref.	Ref.	Ref.			
Female	1136	306 (26.9)	1.44	1.04, 1.99	2.22	0.026	4.93	1	0.026
**Breed**									
Indigenous	1374	342 (24.9)	1.00	Ref.	Ref.	Ref.			
Cross breed	61	28 (45.9)	2.67	1.51, 4.70	3.39	0.001	11.49	1	0.0007
**Age (years)**									
1–2	599	85 (14.2)	1.00	Ref.	Ref.	Ref.			
3	578	198 (34.3)	3.4	2.49, 4.64	7.73	< 0.001			
4–5	258	87 (33.7)	3.31	2.27, 4.82	6.25	< 0.001	65.91	2	< 0.001
**Animals with history of abortion**									
No	870	226 (26.0)	1.00	Ref.	Ref.	Ref.			
Yes	12	7 (58.3)	4.13	1.19, 14.41	2.23	0.026	4.96	1	0.026
**Animal source**									
Born within herd	1383	339 (24.5)	1.00	Ref.	Ref.	Ref.			
Introduced into the herd	52	31 (59.6)	5.08	2.74, 9.44	5.15	< 0.001	26.51	1	< 0.001
**Animal sampled from district with history of RVF outbreaks**									
No	691	136 (19.7)	1.00	Ref.	Ref.	Ref.			
Yes	744	234 (31.5)	1.88	1.41, 2.51	4.29	< 0.001	18.42	1	< 0.001
**Animal with diarrhoea**									
No	1372	353 (25.7)	1.00	Ref.	Ref.	Ref.			
Yes	63	17 (27.0)	0.94	0.45–1.96	-0.15	0.878	0.02	1	0.878
**Animal with nasal discharge**									
No	1418	366 (25.8)	1.00	Ref.	Ref.	Ref.			
Yes	17	4 (23.5)	1.12	0.35–3.66	0.19	0.846	0.04	1	0.846
**Cattle density (heads per square km)**									
≤19	691	126 (19.7)	1.00	Ref.	Ref.	Ref.			
>19–30	500	142 (28.4)	1.61	1.18–2.21	2.96	0.003			
>30	244	92 (37.7)	2.52	1.72–3.68	4.77	< 0.001	24.31	2	< 0.001
**Goats density (heads per square km)**									
≤14	715	144 (20.1)	1.00	Ref.	Ref.	Ref.			
>14–25	477	134 (28.1)	1.54	1.13–2.11	2.72	0.007			
>25	243	92 (38.9)	2.36	1.63–3.42	4.53	< 0.001	21.83	2	< 0.001
**Sheep density (heads per square km)**									
≤2	691	136 (19.7)	1.00	Ref.	Ref.	Ref.			
>2–8	501	142 (28.3)	1.64	1.20–2.23	3.10	0.002			
>8	243	92 (37.9)	2.44	1.68–3.55	4.69	< 0.001	24.05	2	< 0.001
**Feeding practices**									
grazing only	1210	312 (25.8)	1.00	Ref.	Ref.	Ref.			
stall-feeding combined with grazing	225	58 (25.8)	1.04	0.68–1.57	0.17	0.865	0.03	1	0.865

OR, odds ratio; CI, confidence interval; Ref, reference group; SE, Standard error; DE, Degrees of freedom

* The effect of potential risk factors in the univariable mixed-effects logistic regression model

### Multivariable Mixed Effects Logistic Regression Analysis

The final multivariable mixed-effects logistic regression model to examine the effect of potential risk factors on RVFV seropositivity in domestic ruminants included soil water holding capacity properties in the sampled area, age of livestock and animal source into the herd (Table 4). Animals sampled from the villages within the districts with soils of good water holding capacity had higher odds of RVFV seropositivity than animals sampled in the areas with soils of poor water holding capacity (OR = 1.97; 95% CI: 1.58, 3.02; p< 0.001). The odds of seropositivity increased with increasing age, in that compared with animals aged 1–2 years old, those aged 3 and 4–5 years old had 3.40 (CI: 2.49, 4.64; p< 0.001) and 3.31 (CI: 2.27, 4.82, p< 0.001) times the odds of seropositivity, respectively. The domestic ruminants which had been introduced into the herd had significantly higher odds of seropositivity than animals born within the herd (OR = 5.08, CI: 2.74, 9.44; p< 0.001). There was no evidence of collinearity or effect modification between these risk factors and no confounding effect was detected. The intra-cluster correlation coefficient expressing the within-herd clustering of variation was 0.06. The assessment of the predictive accuracy of the final multivariable model based on the area under the curve (AUC) derived from the receiver operating characteristic curve analysis (AUC = 0.68) suggested that the model provided a moderate degree of discrimination.

**Table 4:** Risk factors for RVFV seropositivity in Tanzania included in final multivariable mixed effects logistic regression model (herd was included as random effect variable).

Variable	OR	95% CI	z value	p value	* Pearson Chi square	*DF	*p value
**Water holding capacity of soils in the study areas**							
Poor	1.00	Ref.	Ref.	Ref.			
Good	1.97	1.58, 3.02	4.73	< 0.001	22.36	1	< 0.001
**Age (years)**							
1–2	1.00	Ref.	Ref.	Ref.			
3	3.40	2.49, 4.64	7.73	< 0.001			
4–5	3.31	2.27, 4.82	6.25	< 0.001	65.91	2	< 0.001
**Animal source**							
Born within herd	1.00	Ref.	Ref.	Ref.			
Introduced into the herd	5.08	2.74, 9.44	5.15	< 0.001	26.51	1	< 0.001

OR, odds ratio; CI, confidence interval; Ref, reference group; SE, Standard error; DE, Degree of freedom

* The effect of potential risk factors in the multivariable mixed-effects logistic regression model.

## Discussion

Tanzania has not reported any RVF outbreak since the last epidemic in 2006–2007. The study reported here provides valuable serological information on RVFV activity in domestic ruminants during the IEP in Tanzania. The detected levels of IgG seroprevalence for RVFV varied between study villages in the two Rift Valley ecosystems. The findings of this study suggest that RVFV seropositivity in the study areas is associated with multiple factors including water holding capacity of the soils, age of the animal and animal source. These results need to be interpreted taking into account that the six study villages cannot be considered to be representative of the variation in RVFV risk in Tanzania, in the two Rift Valley ecosystems or in the districts.

Based on the difference in prevalence, it appears that the risk of RVFV seropositivity is higher in animals sampled from Malambo (Ngorongoro district), Ninchoka (Serengeti district) and Chamae (Kongwa district) villages within the eastern Rift Valley ecosystem than animals sampled from Bukirilo (Kibondo district), Nyakasimbi (Karagwe district) and Kajunjumele (Kyela district) villages within the western Rift Valley ecosystem. While the salient reasons for this spatial variation are poorly understood, it is likely to be due to multiple factors, including environmental, livestock density and cultural differences. While the six villages are not representative of the two Rift Valley ecosystems, there are still several ecological characteristics which are typical for these systems and therefore also apply to the study villages. The eastern Rift Valley ecosystem experiences a bimodal pattern of rainfall and the dominant soil types in this region have characteristic features of good water holding capacity compared with western Rift Valley ecosystem which experiences the unimodal rainfall pattern and the dominant soil types in this region are of poor water holding capacity [*50*]. Within the eastern Rift Valley ecosystem pastoralism is characterised by higher livestock density than the western ecosystem. The bimodal rainfall pattern and presence of soils with properties of good water holding capacity in the eastern Rift Valley ecosystem are likely to provide suitable habitat for mosquito breeding and survival, and long term availability of pastures and water for livestock keeping in this region compared with the western Rift Valley ecosystem. The risk of transmission of RVFV can be high and widespread in areas which experiences excessive rainfall and flooding [*1, 2*] which are characteristics of the eastern Rift Valley ecosystem.

The soil types predominant in the districts with the study villages where higher RVFV seroprevalence was recorded were phaezoms (Ngorongoro district), alisols (Serengeti district), fluvisols (Kyela district) and luvisols (Kongwa district). These soil types have high activity of clays and loam texture, making them less vulnerable to erosion and are therefore classified as soils with good water holding capacity properties [*50*]. Their slowly permeable subsoil characteristic subject these soil types to periodic water stagnation and are therefore likely to support mosquito breeding activity and pasture for livestock keeping over longer periods. It should be noted that three of these four districts (Ngorongoro, Serengeti, and Kongwa), are located within the eastern Rift Valley ecosystem and had reported RVF outbreaks in the past. On the other hand, ferralsols are the predominant soils in Kibondo and Karagwe districts in the western Rift Valley ecosystem where lower RVFV seroprevalence was recorded in the study villages. Ferralsols are classified as soils with poor water storage characteristics; their subsoil is overlaid by loamy sand or coarser textures, making them vulnerable to erosion during periods of heavy rainfall [*50*]. As a result, these soils are likely to provide less support to mosquito breeding activity and pasture for livestock keeping.

Of the six studied villages, the seroprevalence was highest (above 37%) in Chame (Kongwa district) and Malambo (Ngorongoro district), both in the eastern Rift Valley ecosystem. Whereas Nyakasimbi (Karagwe district) and Bukirilo (Kibondo district) villages are situated in hilly terrain, Ninchoka (Serengeti district), Malambo (Ngorongoro district), Chamae (Kongwa district) and Kajunjumele (Kyela district) villages are in mainly flat terrain susceptible to flooding during extended periods of heavy rainfall. Although both Kajunjumele (Kyela district) and Bukirilo (Kibondo district) villages experience the long unimodal rainfall pattern staring from October to May, the observed variation in the seropositivity could be partly explained by differences in the terrain and water holding characteristics of the soils between the study areas. The areas with flat terrain are more likely to remain wet and support mosquito breeding and survival, and pasture/water for livestock over longer periods in contrast to those with hilly terrain. It is worth noting that livestock density in Bukirilo (Kibondo district) and Nyakasimbi (Karagwe district) villages, which had never reported RVF outbreak, is lower than the livestock density in the villages with reports of RVF outbreaks (Table 1).

Although both Serengeti and Ngorongoro districts border the SENAPA and NCA ecosystem, respectively, the interesting characteristic is that livestock keeping and trade, and human habitation are allowed within NCA but not within SENAPA. This uniqueness together with uncontrolled trans-boundary animal movements could partly explain the observed differences in the seropositivity between these two different ecosystems, thus supporting the hypothesis that human-wildlife-livestock interaction increases the risk for RVFV transmission potential. Wild animals have been reported to be infected with RVFV [*51*]. Further studies are needed to assess the impact of this complex and local epidemiological situation of human-wildlife-livestock interaction on the risk of RVFV transmission in this area. In contrast, Kyela, Karagwe and Kibondo districts, which are at the country’s border, have never reported RVF outbreaks. In other locations in Tanzania RVFV seropositivity without clinical disease has also been reported in domestic ruminants in Kilombero [*36*] and Kibondo [*52*] districts. Moreover, evidence of RVFV seropositivity without clinical disease during the IEP has been reported in humans in Kyela district [*38*] and in Tanga region [*37*] in Tanzania. Comparable serological prevalence of RVFV antibodies without clinical disease in domestic ruminants born during the IEP has also been reported in Kenya [*53*] and Mozambique [*54*], and in humans in Gabon [*55*].

In this study, a larger proportion of introduced domestic ruminants was recorded in Malambo village in Ngorongoro district compared with the other villages. Furthermore, in Malambo village the introduced animals had significantly higher odds of RVFV seropositivity than in the other districts. However, we were unable to determine whether introduced animals had become infected prior or post introduction to the herd. Local persistence of RVFV infection over time in Ngorongoro district has been suggested in a previous study [*35*]. But this study did not differentiate between locally sourced and introduced animals. Introduction of RVFV to new areas has been linked to livestock movements in Egypt [*56*] and Arabian Peninsula [*57*].

It is unclear why the RVFV seroprevalence reported in this study was not associated with detectable clinical cases in animals. As an example, the Kajunjumele village in Kyela district lies at an altitude of 452m in the flood plains of Lake Nyasa. It receives a total annual rainfall of up to 3000mm and is subjected to regular flooding during the rainy season. Rice cultivation is the main agricultural activity in this area and this will provide abundant mosquito breeding places. This is an area where combined grazing and stall feeding are practiced. The practice of cattle owners in this area to secure their animals at the doorsteps of their houses at night for fear of theft is likely to provide a pathway for RVFV transmission to humans [*38*]. The livestock RVFV seroprevalence of 25.8% reported in this village is comparable to the human seroprevalence of 29.3% reported previously in the same village [*38*]. However, it is surprising that this area had never reported clinical RVF disease in livestock or humans. RVF might be presenting as sporadic cases that are misdiagnosed as other disease conditions, especially in situations where there is no active surveillance and poor diagnostic capacity. Alternatively, demonstration of RVFV seropositivity without clinical disease in all tested animal species suggests that these animal species might be tolerant to clinical disease, had mild infection or that less virulent strains may be circulating in the study areas, which requires further investigation.

Detection of RVFV seroprevalence across all age groups of animals tested supports the notion of endemicity and provides evidence of past undetected but potentially significant local transmission of RVFV. Seropositivity increases with increasing age. A recent serosurvey in Mozambique reported similar levels of RVFV seropositivity without signs of clinical disease in older compared with younger age groups of domestic ruminants [*54*]. Due to their longer productive life compared with sheep and goats, cattle represented the largest proportion of older domestic ruminants sampled in our study. In our study we sampled animals that were born after the last disease outbreak (aged between one and five years old), and therefore the seroprevalence reported here is likely to be a reflection of cumulative exposure to RVFV over time, and long persistence of IgG specific to RVFV in the animals over years. Increased herd immunity resulting from regular exposure to RVFV and long term persistence of antibodies specific to RVFV has been reported previously in domestic ruminants in Kenya [*53*] and Senegal [*58*].

In the current study, immunoglobulin M (IgM) antibodies specific to RVFV were not detected in any of the samples. This suggests that there was no detectable level of active infection at the time of sampling during the dry season, at least in the six villages examined here. This explanation is supported by the fact that IgM has been reported to persist for only 6 to 8 weeks after initial infection [*41*]. Although less well studied, it has been suggested that IgM disappears in 50% of infected animals after 45 days and is absent in 100% of infected animals by 120 days post infection [*59*]. However, RVFV IgG antibodies are believed to persist in animals for life following infection, and therefore its detection provides a reliable index of previous exposure to RVFV [*53, 58*].

It is notable that RVFV seropositivity was found in all six villages, two (Malambo in Ngorongoro district and Ninchoka in Serengeti district) of which are situated in districts close to the border with Kenya and one each in the districts close to the border with Rwanda (Nyakasimbi in Karagwe district), Burundi (Bukirilo in Kibondo district) and Malawi (Kajunjumele in Kyela district). One village (Chamae) is situated in Kongwa district, which is in central Tanzania along the network of major roads. Kibondo, Karagwe and Kyela districts are at the periphery of the national network of major roads. Ngorongoro and Serengeti districts are well integrated into the national network of major roads, facilitating transport of livestock from the Mara to the Arusha region through NCA and SENAPA. Kongwa district is at the centre of Tanzania’s network of major roads facilitating long distance transport of domestic ruminants. Overall, in our study the risk of RVFV seropositivity was higher for the villages from the districts that are more central to the national network of major roads than those from the districts which are at the periphery of the national network of major roads. While we recognise that our very small sample of six villages requires caution in relation to the generalised inferences, we believe that our findings suggest the possibility that the relative position of an area to the network of major roads influences the risk of RVF occurrence. Although the role of animal transport movements in the spread of RVF is poorly understood, it is can be hypothesized that these movements which will be more frequent along major roads offer the potential risk for geographical dispersal of RVFV and/or its vectors. While this still is only a hypothesis, it would be sensible to study the spatial as well as temporal trade patterns of livestock within the country and across the borders. This data will not only be of relevance for RVFV but also for other infectious pathogens.

When interpreting the findings from this study, several limitations need to be taken into consideration. First, due to limited available resources, only six study villages were selected based on a purposive selection process, involving a combination of data generated by a predictive model and knowledge of local veterinarians and farmers in relation to RVF risk. No villages were selected which were considered to not be at risk. This means it will not be possible to draw generalizable inferences from the results obtained in the six study villages to other parts of Tanzania. But the data are suitable for generating hypotheses which should then be investigated further. Second, although fewer animals were reported to have been sourced from outside a particular village, it was, however, not possible to establish the exact source of these animals especially for those purchased at auction markets, and whether they were introduced as infected or susceptible animals. Third, it was challenging to establish whether animals that were purchased at auction markets outside a village’s district might actually have originated from the district the sampled animal was from. Fourth, due to the persistence of IgG in the animal host over several years, it was not possible to estimate the likely period of exposure. It needs to be noted that the last disease outbreak in the study area had occurred in 2006–2007, and it was a requirement for selection that the animals had been born after that outbreak. Fifth, for logistic reasons the virus neutralization test was not performed which could have improved the diagnostic information in this study. Sixth, the findings of this study should be interpreted cautiously since the validity of some of the interview-based data might have been affected by recall bias amongst the animal owners and/or herders.

## Conclusion and Recommendations

Despite the absence of reports of severe RVF outbreaks in the western Rift Valley ecosystem, there is evidence to suggest that RVFV has been circulating in this region over the past years. This study has identified RVFV seropositivity in cattle, sheep and goats sampled from six villages in both the eastern and western Rift Valley ecosystem in Tanzania. However, the animals sampled from villages within the districts in the eastern Rift Valley ecosystem had significantly higher odds of seropositivity than those from villages within the western Rift Valley ecosystem. The sampled animals were born after the last disease outbreak in 2006–2007 suggesting IEP transmission of RVFV was occurring in all study villages. This study explored potential risk factors associated with RVF seropositivity in the six villages. Although the findings of this study suggest that, all sampled domestic ruminants were at risk of being infected with RVFV and that the risk of seropositivity increased with increasing animal age, the risk of seropositivity was higher in crossbreed than indigenous domestic ruminants. A higher level of seropositivity in the three study villages from the eastern than western Rift Valley ecosystems can partly be explained by the differences in climate, geographical and livestock density between the two ecosystems. The three villages in the eastern Rift Valley ecosystem experience a bimodal pattern of rainfall and the dominant soil types in this region have good water holding capacity compared with the three study villages in the western Rift Valley ecosystem which experience a unimodal rainfall pattern and the dominant soil types in this region are of poor water holding capacity. Livestock density is higher in the districts where the study villages are located in the eastern than in those in the western Rift Valley ecosystem. Recognising that the villages are not representative, these findings will make a contribution towards improved predictions of RVF risk in the country.

Future RVF research could focus on several aspects. One would be to establish whether RVF viruses circulating in the eastern and western Rift Valley ecosystems are identical using molecular methods. Furthermore, if they are related, the mechanism of dispersal of RVFV from the eastern Rift Valley to the western Rift Valley ecosystem needs to be studied, in particular the potential role of animal movement and competent mosquito vectors. Given that RVF is a zoonotic disease, public health risk management in Tanzania should adopt integrated approach in the collection of livestock and human data. Research needs to be conducted to establish contact rates between human, livestock and mosquito vectors to better understand the transmission dynamics of the disease, so that more effective control methods can be developed.

## Supporting Information

S1 ARRIVE Checklist ARRIVE Guidelines Checklist. (PDF)

S1 Table Range of Inhibition and IgM detection ELISA values for domestic ruminant serum samples tested for antibodies specific to RVFV (DOC)

S1 Text Questionnaire. (DOC)
