# Supplementary material for: A Spatial Analysis of Rift Valley Fever Virus Seropositivity in Domestic Ruminants in Tanzania
Source: PLoS One. 2015 Jul 10;10(7):e0131873. doi: 10.1371/journal.pone.0131873 (PMC4498811; doi:10.1371/journal.pone.0131873)
Supplement: S1 Table — (DOC) [file pone.0131873.s002.doc]

**S1 Table.** Range of Inhibition and IgM detection ELISA values for domestic ruminant serum samples tested for antibodies specific to RVFV

| **Animal** | **Inhibition ELISA** | | **IgM ELISA** |
| --- | --- | --- | --- |
|  | **Negative** | **Positive** | **Negative** |
| **Goat** | -4.96988-41.30795 | 58.41577-100.92829 | -1.04361-3.98323 |
| **Sheep** | -0.71713-32.95583 | 48.22052-99.85740 | -2.53646-5.88713 |
| **Cattle** | -9.80570-38.36193 | 53.00948-104.86056 | -1.34178-6.107504 |
