# Supplementary material for: A Spatial Analysis of Rift Valley Fever Virus Seropositivity in Domestic Ruminants in Tanzania
Source: PLoS One. 2015 Jul 10;10(7):e0131873. doi: 10.1371/journal.pone.0131873 (PMC4498811; doi:10.1371/journal.pone.0131873)
Supplement: S1 Text — (DOC) [file pone.0131873.s003.doc]

**S1 Text.** Questionnaire

**A). Study area data**

**Geographic Coordinates of the village (in Decimal Degrees)**

| **Latitude** |  |  |  |  |  |  |  |  | **Longitude** |  |  |  |  |  |  |  |  |
| --- | --- | --- | --- | --- | --- | --- | --- | --- | --- | --- | --- | --- | --- | --- | --- | --- | --- |

| (a).Rift Ecosystem  0= Western 1=Eastern | (b).Region | (c).District | (d).Village | (e).Village elevation (metres above sea level) | (f).Predominant Soil  type in the district | (g). Total average monthly rainfall in the district (mm) | (h). Total average annual rainfall in the district (mm) |
| --- | --- | --- | --- | --- | --- | --- | --- |
|  |  |  |  |  |  |  |  |

| (i).Rainfall pattern in the district  0=Unimodal 1=Bimodal | (j).District has reported outbreak  0=No 1=Yes | (k).Cattle density in the district (heads per square km) | (l).Goats density in the district (heads per square km) | (m).Sheep density in the district (heads per sqaure km) |
| --- | --- | --- | --- | --- |
|  |  |  |  |  |

**B). Animal level data**

**Geographic Coordinates of the herd (in Decimal Degrees)**

| **Latitude** |  |  |  |  |  |  |  |  | **Longitude** |  |  |  |  |  |  |  |  |
| --- | --- | --- | --- | --- | --- | --- | --- | --- | --- | --- | --- | --- | --- | --- | --- | --- | --- |

| (a).Animal species 1=Caprine 2=Ovine 3=Bovine | (b).Animal feeding options 1=grazing oly 2=combined stall-feeding and grazing | (c).No. Animal species in herd 1=One 2=Two 3=Three | (d).Animal age (years) | (e).Animal breed 1=Indegen-ous 2=Cross breed | (f).Animal source to herd in the district 1=Born 2=Introduced | (g).Animal sex 1=Male 2=Female | (h).Abortion past 12months: 1=No  2=Yes 3=Don’t know  4=Not Applicable | (i).Body temperature(^o^C) on the date of herd vist 1=Normal {37.8-40} 2=Above normal (>40) | (j).Any ill health on the day of herd visit  1=No  2=Yes (if yes; describe) | (k).Blood sample ID.No. | (l).Laboratory results:  RVFV serostatus 0=negative 1=positive |
| --- | --- | --- | --- | --- | --- | --- | --- | --- | --- | --- | --- |
|  |  |  |  |  |  |  |  |  |  |  |  |
|  |  |  |  |  |  |  |  |  |  |  |  |
|  |  |  |  |  |  |  |  |  |  |  |  |
|  |  |  |  |  |  |  |  |  |  |  |  |
|  |  |  |  |  |  |  |  |  |  |  |  |
|  |  |  |  |  |  |  |  |  |  |  |  |
|  |  |  |  |  |  |  |  |  |  |  |  |
|  |  |  |  |  |  |  |  |  |  |  |  |
|  |  |  |  |  |  |  |  |  |  |  |  |
|  |  |  |  |  |  |  |  |  |  |  |  |
|  |  |  |  |  |  |  |  |  |  |  |  |
|  |  |  |  |  |  |  |  |  |  |  |  |
|  |  |  |  |  |  |  |  |  |  |  |  |
|  |  |  |  |  |  |  |  |  |  |  |  |
|  |  |  |  |  |  |  |  |  |  |  |  |
|  |  |  |  |  |  |  |  |  |  |  |  |
|  |  |  |  |  |  |  |  |  |  |  |  |
|  |  |  |  |  |  |  |  |  |  |  |  |
|  |  |  |  |  |  |  |  |  |  |  |  |
|  |  |  |  |  |  |  |  |  |  |  |  |
